# Life cycle assessment of edible insects (*Protaetia brevitarsis seulensis* larvae) as a future protein and fat source

**DOI:** 10.1038/s41598-021-93284-8

**Published:** 2021-07-07

**Authors:** Amin Nikkhah, Sam Van Haute, Vesna Jovanovic, Heejung Jung, Jo Dewulf, Tanja Cirkovic Velickovic, Sami Ghnimi

**Affiliations:** 1 Department of Food Technology, Safety and Health, Faculty of Bioscience Engineering, Ghent University, Coupure Links 653, 9000 Ghent, Belgium; 2 Department of Environmental Technology, Food Technology and Molecular Biotechnology, Ghent University Global Campus, Incheon, South Korea; 3 Faculty of Chemistry, Centre of Excellence for Molecular Food Sciences, University of Belgrade, Belgrade, Serbia; 4 Research Group Sustainable Systems Engineering (STEN), Department of Green Chemistry and Technology, Ghent University, Coupure Links 653, 9000 Ghent, Belgium; 5 Serbian Academy of Sciences and Arts, Belgrade, Serbia; 6 CNRS, LAGEPP UMR 5007, Université Claude Bernard Lyon 1, 43 Bd 11 Novembre 1918, 69622 Villeurbanne, France; 7 ISARA Lyon, 23 Rue Jean Baldassini, 69364 Lyon Cedex 07, France

**Keywords:** Climate sciences, Environmental sciences

## Abstract

Because it is important to develop new sustainable sources of edible protein, insects have been recommended as a new protein source. This study applied Life Cycle Assessment (LCA) to investigate the environmental impact of small-scale edible insect production unit in South Korea. IMPACT 2002 + was applied as the baseline impact assessment (IA) methodology. The CML-IA baseline, EDIP 2003, EDP 2013, ILCD 2011 Midpoint, and ReCiPe midpoint IA methodologies were also used for LCIA methodology sensitivity analysis. The protein, fat contents, and fatty acid profile of the investigated insect (*Protaetia brevitarsis seulensis* larvae) were analyzed to determine its potential food application. The results revealed that the studied edible insect production system has beneficial environmental effects on various impact categories (ICs), i.e., land occupation, mineral extraction, aquatic and terrestrial ecotoxicity, due to utilization of bio-waste to feed insects. This food production system can mitigate the negative environmental effects of those ICs, but has negative environmental impact on some other ICs such as global warming potential. By managing the consumption of various inputs, edible insects can become an environmentally efficient food production system for human nutrition.

## Introduction

Owing to the increasing demand for meat, there is a need for discovering alternative sources of protein^1^. Moreover, many studies have shown that most common food production systems, such as beef^2,3^, chicken^4–6^, pork^7,8^, fish^9,10^, and plant-based products (bean, corn, soybean, and wheat)^11,12^ are not environmentally efficient, and that the existing production systems of protein sources have enormous environmental disadvantages.

Hence, the development of new alternative sustainable food sources is very important. In recent years, insects have been recommended as a novel food source for human consumption and the amount of insect production units has been increasing worldwide. Insect production can be considered as a solution for two problems, namely, the increasing demand for food by the production of edible insects, and waste management by composting food waste^13^. The following sections discuss the current status of edible insect production, small-scale insect production systems, and the literature review on sustainability assessment of edible insect production.

### Current status of edible insect production

Approximately 2000 insect species are consumed as food around the world, particularly in tropical countries^1^. Based on the European Food Safety Scientific Committee report, nine different insect species are currently recorded as being farmed for feed and food production^14^. For instance, there are approximately 20,000 cricket farms in Thailand, which produce 7500 metric tons of insects per year which are used for domestic consumption and the rest for market^15^. Currently, two billion people across the world eat insects, and insects are being consumed as food in approximately 80 countries^16^.

In South Korea (one of the main consumers), edible insects were previously more widespread in the populations’ nutrition. Under the economic development plan implemented in the 1970s, the production and also the consumption of edible insects decreased. In recent years, the consumption is on the rise again, and the value of the edible insect market in south Korea has increased from 143 million in 2011 to 259 million in 2015^17^.

There are many small-scale edible insect startups and production units around the world. For example, most insect producers in Thailand are small and medium size enterprises which require relatively low land usage and capital investment^18^. Moreover, environmental issues are a major factor with regard to the sustainable development of food production systems. Thus, this study applied Life Cycle Assessment (LCA) to estimate the environmental impact of small-scale edible insect production in South Korea, as an example of small-scale edible insect production and the nutritional value was assessed with a focus on proteins and fats.

### Literature review

Various studies have been conducted with regard to the LCA of the production of insects for use as feed^19–22^. However, in recent years, researchers have focused on the environmental life cycle impacts of edible insect production systems. Table 1 shows the exemplary studies on the LCA of edible insect production systems to demonstrate variations in the scope, impacts assessments and results of the studies. Oonincx and De Boer^23^ compared the protein production from two species of mealworms (*Tenebrio molitor* and *Zophobasmorio*) with conventional sources of protein like beef, milk, chicken, and pork. The results revealed that lower GHG emissions and land use are required for mealworm production, while the required amount of energy is similar to that of conventional and mealworm protein production systems. Thus, they concluded that mealworms are a more sustainable source of edible protein. Halloran et al.^14^ compared the environmental impact of cricket farms to that of chicken farms in Thailand. The results revealed that protein from insects is more environmentally efficient compared with that of chicken. One study reported that the direct CO_2_eq emissions from bio-waste conversion using insects (black soldier flies) were 47 times lower compared with the emissions produced by an open windrow composting facility^24^. Smetana et al.^25^ reported that the insect biomass is twice more environmentally efficient compared with that of chicken meat.

**Table 1:** Exemplary studies on LCA of edible insect-based food.

Insect species	The studied region	Functional unit	Impact assessment methodology	Focus of the research	Environmental hotspots	Reference
*Hermetia illucens* (Black Soldier Fly)	Germany	One kg of dried defatted insect powder and 1 kg of ready for consumption fresh product without packaging	ReCiPeV1.08 and IMPACT 2002 +	Compare insect-based food product with other food products	Feed production	Smetana et al.^26^
*Hermetia illucens* (Black Soldier Fly)	Italy	One tonne of food waste, one kg of protein and lipid	CML 2 baseline 2000, IPCC 2007, Cumulative energy demand method, and CML 2001	Food waste bioconversion by insect	Electricity consumption and transportation	Salomone et al.^13^
Mealworm (*Tenebrio molitor* and *Zophobas morio*)	Netherlands/Finland	One kg of mealworms	Not available	GWP of the future potential industrial scale	Feed crop production and direct heating energy	Joensuu and Silvenius^27^
*Gryllusbimaculatus De Geer* (field cricket) and *Acheta domesticus* (house cricket)	Thailand	One kg of edible mass and one kg of protein	ILCD	Comparing environmental impacts of insect with chicken	Feed production	Halloran et al. ^14^
*Hermetia illucens* (Black Soldier Fly)	Indonesia	One tonne of bio-waste	IPCC 2013 100a and ReCiPe Midpoint Hierarchist (H)	Bio-waste conversion using insect	Electricity consumption	Mertenat et al.^24^
*Hermetia illucens* (Black Soldier Fly)	Netherlands	One kg of dried and pelletized organic fertilizer, one kg of fresh insect used as pet food, one kg of protein, and one kg of insect fat used as feed	IMPACT2002 +	Sustainably of insect production as feed and food	Feed production and energy use	Smetana et al.^25^
Black soldier fly (*Hermetia illucens*) and mealworm (*Tenebrio molitor*)	Germany	One kg	IMPACT2002 +	Insect margarine	Raw materials consumption	Smetana et al. ^28^
*Protaetia brevitarsis seulensis*	South Korea	One kg of dried insect, protein, and fat	CML-IA baseline, EDIP 2003, EDP 2013, ILCD 2011 Midpoint, ReCiPe midpoint, and IMPACT 2002 +	*Protaetia brevitarsis seulensis* larvae as a future protein and fat source	–	Current Study

As an example, this study investigated the environmental sustainability of small-scale edible insect production in South Korea using LCA methodology. The current study is the first research on the environmental impacts of *Protaetia brevitarsis seulensis* (PBS). In addition, fatty acid (FA) profile, protein and fat contents of PBS were determined to assess the nutritive value of PBS edible insect.

## Results and discussion

### Protein and fat content of *Protaetia brevitarsis seulensis*

Because one of the objectives of this study was to compare the environmental effects of insect protein with those of protein produced from conventional human nutrition sources, the protein content of larvae was determined and the LCA results are expressed per kg of insect protein. The protein and fat contents of the dried larvae of PBS were determined as 50.5% and 13.5%, respectively. The results are in good agreement with the results of previous studies, and revealed that the dried larvae contained more than 50% protein and between 10 and 25% fat^29,30^. The insects’ protein content is generally similar to that of beef, pork, and chicken, and contains more polyunsaturated fatty acid with higher contents of various minerals, such as zinc and iron^15^. PBS *larvae* is one of the five types of dried edible insects that are currently available in the Korean market. The larvae stage of PBS is also currently being used in traditional Chinese medicine^31^ because it produces therapeutic effects for the treatment and prevention of various types of diseases (inflammatory disease, liver cirrhosis, and hepatitis) and cancers (hepatic and breast cancer)^32,33^. Therefore, in the near future, it can be used as a potential source of protein and fat.

The GC–MS (Gas Chromatography Mass Spectrometry) analysis of the fatty acid profile revealed the presence of 18 FAs, whose spectra overlap with the spectra from the NIST base (Table 2) with a probability of more than 93%. Amongst the 18 identified FAs, six of them were saturated FAs (SFA) (two of them has an odd number of C atoms:C15:0 and C17:0); eight were unsaturated FAs (four monounsaturated FAs (MUFA) and four polyunsaturated FAs (PUFA)); four were methyl FAs. The most abundant FA was oleic acid (60.38%), which along with palmitic, palmitoleic, and linoleic acid contributed to 90% of the total FA content. These results are in agreement with the results reported by Yeo et al.^29^.

**Table 2:** Fatty acid profile of *Protaetia brevitarsis seulensis* larvae; values are expressed as a mean value ± SD.

	Retention time (min)	Fatty acid		Content (% of total fatty acids)
1	9.720	Myristic acid	C14:0	0.58 ± 0.003
2	10.200	13-Methyltetradecanoic acid	C14:0 13 methyl	1.28 ± 0.001
3	10.664	Pentadecanoic acid	C15:0	0.11 ± 0.005
4	11.200	14-Methylpentadecanoic acid	C15:0 14 methyl	0.49 ± 0.034
5	11.718	Palmitic acid	C16:0	16.28 ± 0.143
6	12.148	Palmitoleic acid	C16:19c	8.32 ± 0.008
7	12.250	11-cis-Hexadecenoic acid	C16:111c	1.17 ± 0.052
8	12.352	15-Methyhexadecanoic acid	C16:0 15 methyl	0.50 ± 0.018
9	12.550	14-Methylhexadecanoic acid	C16:0 14 methyl	0.27 ± 0.018
10	12.900	Heptadecanoic acid	C17:0	0.10 ± 0.012
11	13.300	6-cis-9-cis-12-cis-Hexadecatrienoic acid	C16:36c9c12c	0.31 ± 0.010
12	14.200	Stearic acid	C18:0	1.69 ± 0.004
13	14.600	Oleic acid	C18:19c	60.38 ± 0.021
14	14.725	11-cis-Octadecenic acid	C18:111c	1.83 ± 0.023
15	15.317	Linoleic acid	C18:29c12c	4.92 ± 0.056
16	15.800	γ-Linolenic acid	C18:36c9c12c	0.19 ± 0.004
17	17.000	Arachidic acid	C20:0	0.50 ± 0.003
18	19.050	Arachidonic acid	C20:45c8c11c14c	0.31 ± 0.007

The contribution of MUFA to the total FA content was 71.70%, which resulted in a very high MUFA value in the calculated SFA:MUFA:PUFA ratio of 3.3:12:1.This exhibits a discrepancy with the recommended SFA:MUFA:PUFA ratio for a healthy nutrition (1.25:1.5:1). Therefore, it can be concluded that fats from PBS are a good source of MUFA. Moreover, according to the literature, it is known that MUFAs promote a healthy blood lipid profile and improve blood pressure, insulin sensitivity, and glycemic control^34–36^.

The effectiveness of PBS larvae in traditional medicine for the treatment and prevention of various diseases (inflammatory disease, liver cirrhosis, and hepatitis) and cancers (hepatic and breast cancer) can be explained by the presence of various FAs, such as palmitic (16.28%), palmitoleic (8.32%), and oleic acid (60.38%) at a very high concentration in the fats of these larvae. Additionally, palmitoleic acid has been associated with increased insulin sensitivity and decreased lipid accumulation in the liver^37^. Yoo et al.^32^ demonstrated that the dichloromethane extract from PBS, which contains FAs (palmitic and olelic acid), has anti-cancerogenic effects. For the first time, our study revealed the presence of four methyl-FAs. Branched-chain FAs are common constituents of bacteria and animal lipids. Amongst them, 13-methyltetradecanoic acid is the most abundant (1.28%), and has been well-known to induce the apoptosis or programmed cell death of certain human cancer cells^38,39^. According to the total fat, protein content, and FA analysis results, it can be concluded that the PBS larvae fed with banana waste can be used as a potential source of protein and fat.

### LCA results

Table 3 presents the characterization indicators of PBS edible insect production in South Korea. The results revealed that the investigated edible insect production system has beneficial environmental effects on certain ICs, such as land occupation, mineral extraction, aquatic and terrestrial ecotoxicity (4 ICs out of 15). In other words, this food production system can mitigate the environmental impacts of the abovementioned ICs, due to utilization of bio-waste (mushroom production waste and banana peels) to feed insects by turning something harmful for environment into compost. Previous studies on the LCA of chicken^4–6^, beef^2,3^, milk^40–42^, and crop production^43^ have shown that the production of these protein sources has negative environmental effects on all investigated ICs.

**Table 3:** Characterization indices of *Protaetia brevitars seulensis* production.

Impact category	Unit	Quantity		
		Per kg biomass	Per kg protein	Per kg fat
Global warming	kg CO_2_ eq	8.05	15.93	59.60
Non-renewable energy	MJ primary	32.46	64.63	241.75
Ozone layer depletion	kg CFC-11 eq	1.58 × 10^−7^	3.12 × 10^−7^	1.17 × 10^−6^
Aquatic eutrophication	kg PO_4_ P-lim	2.76 × 10^−4^	5.46 × 10^−4^	2.04 × 10^−3^
Ionizing radiation	Bq C-14 eq	59.74	118.29	442.49
Carcinogens	kg C_2_H_3_Cl eq	0.05	0.09	0.35
Aquatic acidification	kg SO_2_ eq	0.01	0.01	0.04
Respiratory organics	kg C_2_H_4_ eq	0.001	0.002	0.007
Non-carcinogens	kg C_2_H_3_Cl eq	0.02	0.04	0.15
Terrestrial acid/nutri	kg SO_2_ eq	0.03	0.05	0.20
Respiratory inorganics	kg PM_2.5_ eq	1.68 × 10^−3^	3.33 × 10^−3^	1.25 × 10^−2^
Aquatic ecotoxicity	kg TEG water	− 312.47	− 618.75	− 2314.60
Mineral extraction	MJ surplus	− 0.04	− 0.07	− 0.26
Terrestrial ecotoxicity	kg TEG soil	− 136.46	− 270.21	− 1010.78
Land occupation	m^2^org.arable	− 0.10	− 0.20	− 0.75

Negative values refer to savings and positive values refer to impacts.

Moreover, negative environmental effects on some ICs, namely, ozone layer depletion, non-renewable energy, aquatic eutrophication, ionizing radiation, carcinogens, aquatic acidification, non-carcinogens, respiratory inorganics, respiratory organics, terrestrial acid/nutria, and global warming, have been observed. The environmental impacts of 1 kg of dried insect production on global warming, ozone layer depletion, and renewable energy were calculated as 8.05 kgCO_2_eq, 1.58 × 10^−7^ kg CFC-11 eq, and 32.46 MJ, respectively. Moreover, the values of the abovementioned ICs for 1 kg of protein produced from insects were 15.93 kgCO_2_eq, 3.12 × 10^−7^ kg CFC-11 eq, and 64.63 MJ, respectively. The same values for 1 kg of fat produced from insects were calculated 59.60 kgCO_2_eq, 1.17 × 10^−6^ kg CFC-11 eq, and 241.75 MJ, respectively (Table 3). The GWP of farming 1 kg of insects and producing 1 kg of protein from insects in Thailand has been reported as 4.0 and 3.9 kgCO_2_eq, respectively^14^. The GWP of 1 kg of protein and lipids produced from the *Hermetia illucens* insect has been reported as 2.1 and 2.9 kgCO_2_eq, respectively^13^. Some insect species (*Hermetia illucens* and *Tenebrio molitor*) have been shown a promising potential to be used as an alternative for animal and plant-based lipids products, such as butter or margarine^28^.

The global warming potential of 1 kg protein production form PBS insect (15.93 kgCO_2_eq) was lower than the conventional meat sources, such as chicken (18–36 kgCO_2_eq), pork (21–53 kgCO_2_eq), and beef (75–170 kgCO_2_eq)^44^. Moreover, as it was mentioned earlier, the studied production system has beneficial environmental impacts in 4 out of the 15 studied impact categories which is an advantage compared to the above-mentioned conventional meat production systems. By managing the consumption of various inputs, the PBS edible insect production system can become an environmentally efficient food production system for human nutrition, given its high level of protein content and its potential benefit for environment.

**Figure 1:**
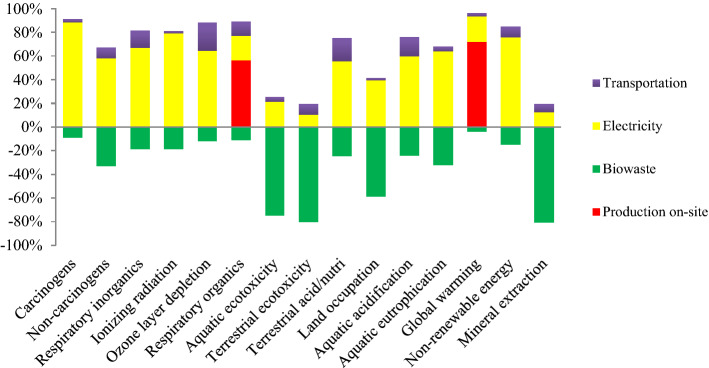
Relative contribution to the environmental impact of *Protaetia brevitars seulensis*.

Figure 1 shows the proportion of inputs in the environmental effects of PBS larvae. The results revealed that the electricity consumption was the environmental point of interest in the production system. The production on-site emissions accounted for the largest proportion of the environmental impact pertaining to global warming and respiratory inorganic ICs. Treatment of bio-waste, which is used to feed insects, exerted beneficial environmental effects on all investigated ICs. In cricket production, the environmental point of interest is related to the feed production^14^. Food wastes have some remarkable nutritional properties that can be valorized for feeding edible insects^45^.

**Figure 2:**
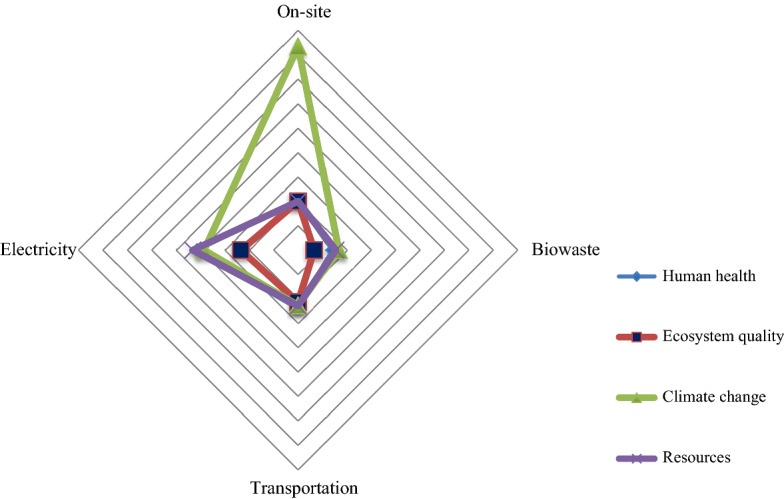
Normalized damage assessment of production system based on different consumption inputs.

**Figure 3:**
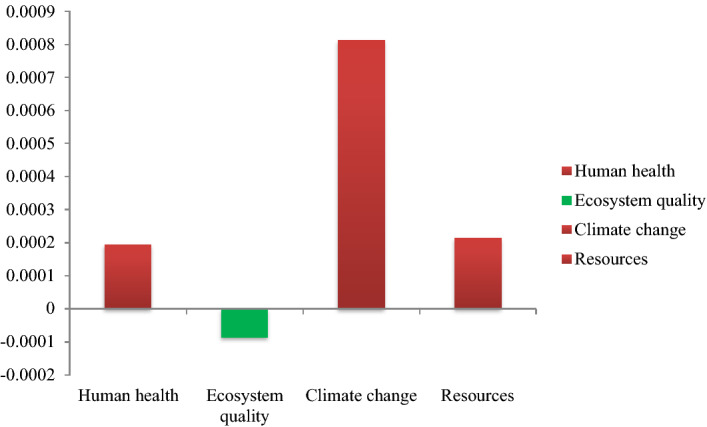
Normalized values of different PBS edible insect damage categories.

Figure 2 shows the normalized damage assessment of the investigated production process in terms of various consumption inputs. The normalized values of damage assessment of the PBS edible insect production are also shown in Fig. 3. The PBS edible insect production system has positive environmental impact within the ecosystem quality damage category; however, it has negative impact on climate change and resource usage, and human health.

The single scores of the damage categories in PBS edible insect production are presented in Table 4. Based on the beneficial environmental impacts of PBS edible insect production in various ICs, edible insects can become an environment efficient food production system for human nutrition by managing certain consumption inputs. In fact, only 40–50% of the produced biomass of cattle, poultry, and pigs are used directly as food. In contrast, the entire body of edible insects can be used as food^46^. Moreover, insects as mini-livestock have many environmental benefits and similar nutritional quality compared with conventional livestock production systems^1^. The primarily studies show that edible insect cell culture also may provide a more cost-efficient platform of cell-based meat system, according to the unique properties of insect cells^47–49^. Edible insects have the potential to be the future food given to their positive nutritional properties and relatively low environmental impacts; however, there are still food safety concerns associated with the consumption of insects, namely, the microbiological and chemical health risk^50,51^. The current study, investigated the environmental impacts (climate change, resource depletion, human health, and ecosystem quality) associated with the PBS production system, and the microbiological and chemical health risk of the final product was not included in the LCA. Further research is needed to look at the microbiological and chemical health risk of this species toward moving to a sustainable edible insect-based production system.

**Table 4:** Single score of damage categories in PBS edible insect production (unit = mPt).

	Bio-waste treatment (avoided activity)	Electricity	Production on-site	Transportation	Total
Human health	− 0.06	0.21	0.0002	0.04	0.19
Ecosystem quality	− 0.13	0.03	0	0.01	− 0.09
Climate change	− 0.03	0.19	0.63	0.03	0.81
Resources	− 0.05	0.23	0	0.03	0.21
Total	− 0.27	0.67	0.63	0.11	1.14

Negative values refer to savings and positive values refer to impacts.

### LCIA methodology sensitivity analysis

The total characterization indices of PBS edible insects are presented in Table 5, as determined using various IA methodologies. These results may help in gaining agreement with the findings of relevant LCA studies on edible insects. Moreover, the results revealed that the global warming potential of farming 1 kg of insects ranges from 8.05 kgCO_2_eq to 12.52 kgCO_2_eq. The amount of ozone layer depletion caused by production of 1 kg of insects ranges from 1.57 × 10^−7^ to 1.58 × 10^−7^ kgCFC-11 eq. The results pertaining to global warming potential was obtained using the IMPACT 2002 + midpoint and is remarkably different to the results obtained using other IA methodologies.

**Table 5:** Characterization indices of PBS edible insects determined using various IA methodologies.

Impact assessment	Global warming (kg CO_2_eq)	Ozone layer depletion (kg CFC-11 eq)
CML-IA baseline	11.49	1.58 × 10^−7^
EDIP 2003	11.42	1.58 × 10^−7^
EDP 2013	11.49	1.58 × 10^−7^
ILCD 2011 Midpoint	11.56	1.57 × 10^−7^
**IMPACT 2002 + **	**8.05**	**1.58 × 10**^−**7**^
ReCiPe midpoint	12.52	–

The base scenario is shown in bold numbers.

## Conclusions

The development of sustainable food production systems is highly important for achieving food security. Moreover, environmental efficiency is one of the main pillars of sustainability. However, many conventional food production systems are not sustainable. Thus, this study investigated the life cycle environmental sustainability of small-scale PBS production. The obtained results revealed that PBS edible insect production systems can be considered as a sustainable food production system owing to their positive environmental effects on 4 out of the 15 investigated ICs. Moreover, according to the total protein and fat, content, and FA analysis results, it can be concluded that the PBS larvae fed with banana waste can be used as a potential source of protein and fat source. However, various negative environmental impacts were observed in some categories. For example, the global warming potential in the production of 1 kg of insects ranged from 8.05 kgCO_2_eq to 12.52 kgCO_2_eq based on the application of different IA methodologies. Finally, the environmental efficiency of the insect production system can be increased by managing certain inputs, such as electricity.

## Materials and methods

**Figure 4:**
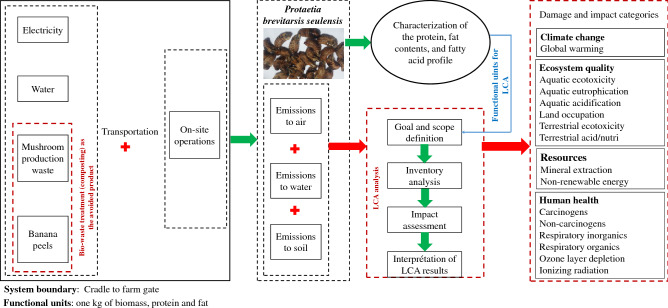
Life cycle assessment procedure of *Protaetia brevitarsis seulensis* production.

Figure 4 shows the life cycle assessment procedure of PBS production. Accordingly, the inputs and yield of PBS edible insects in South Korea were determined. Then, the obtained data were used to conduct cradle-to-gate environmental impact evaluation for the production systems. As a strong and standardized methodology, LCA was used to conduct the environmental consequences of edible insect as a future protein and fat source.

### Insect production system

This study was conducted at Gwangmyeong-si in South Korea, which is located in the Mid-West region of Gyeonggi-do, and at a metropolitan area in Korea (the central part of the Korean Peninsula). The investigated region consists of 38.8% of mountainous area and 28.9% of arable land, where in rice was predominantly cultivated but as the agricultural population decreased, the agricultural activities shifted to the production of high-value vegetable and fruit crops. Gwangmyeong-silies in an average agro-climatic zone in South Korea with four distinct seasons, and average rainfall of 1556 mm per annum; the temperature ranges from an average low of − 1.1 °C to an average high of 25.9 °C.

The investigated insect species is PBS larvae. PBS larvae is one of the five species which are consumed in South Korea^52^. The investigated production unit uses mushroom waste to feed insects, and banana waste to feed immature insects. Temperature is kept around 25 °C throughout the year. However, the relative humidity of the farm was not managed during the process. The volume of the breeding box was 36 L (600 mm × 450 mm × 200 mm). On average, the investigated insects lay eggs every seven to ten days. PBS has four life stages: egg, larva, pupa, and adult. It takes 10 weeks for an egg to become a larva and then it is ready to be collected. The investigated farm was a small insect farm with the capacity of 960 kg larvae (dry basis) production per year. The insects produced in the studied system are available legally for consumption on Korean markets. The investigated insect specious (PBS) is relatively expensive in Korea because of its medicinal properties. Korean food law has limits in PBS larvae on the presence of heavy metals (lead, cadmium and arsenic) and microbial indicators of hygiene/food safety (coliforms, *E. coli*)^53^.

### Sample preparation

Air dried PBS larvae were collected from the insect farm located in Gwangmyeong-si, South Korea. The dried sample was homogenized using mortar and a pestle, and stored in a plastic box at − 20 °C until further analysis.

### Determination of protein and fat content

The fat and protein content of PBS larvae was determined using standard methods. The nitrogen (N) and proteins were investigated by the Kjeldahl method^54^. The protein content was determined through multiplying the N content by the coefficient of 6.25.

The fat content was determined after ethyl ether extraction in a Soxhletapparatus for six hours. Subsequently, the ethyl ether was removed through a rotary evaporator. After that, the extracted sample was weighed until a constant sample weight was reached.

### Fatty acid methyl esters preparation

The FA composition was calculated by the GC/EI-MS of the fatty acid methyl esters (FAME), which were prepared by transmethylation based on the following procedure. In short, 25 mg of dried PBS larvae powder were measured in a Pyrex test tube with a Teflon lined screw cap. Next, 3.3 mL of methanol/hydrochloric acid (2 M in methanol) mixture were added into the tube. After vigorous vortexing for 5 to 10 s, 0.3 mL of chloroform, which contained an internal standard (13:0) and antioxidant (BHT), were added and the tube was tightly sealed. After vigorous vortexing for 30 s, the tube was heated at 90 °C for 2 h. When it gets cooled off to room temperature, the FAME were extracted by adding 0.9 mL of miliQ water into the tube. The mixture in the tube was vortexed for 5 to 10 s, and then 1.8 mL of n-hexane were added and vortexed again for 20 to 30 s. The n-hexane layer containing the FAME was separated by centrifugation for 5 min at 4000 rpm. The uppern-hexane phase was drawn off and transferred to a sample vial for GC/EI-MS analysis. The preparation of FAME was performed in two duplicates.

### Analysis and identification of fatty acids using GC/EI-MS

The analysis of FAME was done according to Ristivojević et al.^55^. In short, an Agilent 6890 gas chromatograph equipped with a DB-23 capillary column (30 m × 0.25 mm id;film thickness of 0.25 μm) was used (Aglient Technologies Inc., Santa Clara, CA, USA). The capillary column was directly joined to an Agilent 5973 mass spectrometer (Agilent Technologies Inc.). The sample (1 μL) was injected into the capillary column with a split ratio of 10:1. Helium (purity of 5.0) was applied as the carrier gas with a flow rate of 0.6 mL/min. The temperatures of the detector and the injector were set to 230 °C and 250 °C, respectively.

The FAME were determined through comparing their retention times with those of the FAME standards (Supelco-37 FAME mix) under the same conditions, and through comparing their mass spectra with those stored in the Mass Spectral Library of the National Institute of Standards and Technology (NIST).

### Objective

The objective of this study was to conduct an attributional life cycle environmental impact analysis of the small-scale PBS edible insect production in South Korea and assess the nutritional value of the investigated insect. Different functional units (FUs) for insect production systems have been considered for LCA, which means that all inputs and impact categories (ICs) in the assessment are normalized. As presented in Table 6, the mass-based FU is commonly used in LCA studies on edible insects. Therefore, in this study, 1 kg of dried insects was selected as the FU. Additionally, 1 kg of protein in insects as well as 1 kg fat were considered as the secondary FUs for comparing the environmental consequences of protein production from insects with other conventional protein and fat sources of human nutrition. The system boundary of this research was the cradle to farm gate insect production system, including inputs (electricity, water, mushroom production wastes, banana peels, and transportation) as well as operations of on-site production (see Fig. 4).

**Table 6:** Main primary inventory data for small-scale *Protaetia brevitarsis seulensis* production.

Inputs–outputs	Unit	Quantity
**Inputs**		
Bio-waste (mushroom waste)	kg	3600
Bio-waste (banana waste)	kg	300
Water	m^3^	324
Electricity	kWh	357
Transportation of bio-waste to insect farm	kg × km	180,000
Transportation of final product	kg × km	12,000
**Outputs**		
Dried insect	kg	120
Compost		
CO_2_	kg	475.2
CH_4_	kg	14.4
N_2_O	kg	1.08

### Inventory analysis

The cradle-to-gate environmental impact for PBS edible insect production was evaluated using LCA. Table 6 presents the main primary inventory data for small-scale edible insect production. The emitted pollutants were classified as the cradle-to-gate (background) and gate-to-gate (foreground) emissions. The emitted pollutants in the background phase (production of input materials) were adapted from the ecoinvent 3.0^56^ database using the SimaPro9.0.0.49^57^ software. The foreground (production on-site) emissions were the pollutants emitted during the composting process, such CO_2_, N_2_O, and CH_4_. The amounts of emitted pollutants during composting process were calculated based on EPA, 2010^58^. Feed is a major factor with regard to the total environmental impacts of insect production either as a burden or as avoided impacts in case of waste treatment^59^. In this study, the emissions within composting process were included as production on-site emissions (Table 6), and the bio-waste treatment was considered as an avoided product. Accordingly, the inventory of cradle to farm gate emissions for one kg PBS insect production is provided as Supplementary 1.

### LCIA methodology sensitivity analysis

The impact assessment (IA) methodologies of previous studies on the LCA of insects are presented in Table 1. The selection of the IA methodology may significantly influence the obtained results of every LCA study on food production systems. In this study, IMPACT 2002 + was employed as the baseline IA methodology owing to its various impact (15 impact categories) and damage categories. IMPACT 2002 + divides the 15 impact categories into four damage categories (endpoints level), i.e., climate change, resource depletion, human health, and ecosystem quality^60^. This IA methodology is the hybrid application of IMPACT 2002, Eco-Indicator 99, CML, and IPCC. Additionally, five other IA methodologies, namely, the ReCiPe midpoint^61^, CML-IA baseline^62^, EDIP 2003^63^, EDP 2013^64^, and ILCD 2011 Midpoint^65^, were also evaluated for comparison with the baseline IA methodology, that is, IMPACT 2002 + . The above-mentioned impact categories were compared in terms of the characterization indices of global warming potential and ozone layer depletion since those are the mutual impact categories considered by the six studied impact assessment methodologies.

## Supplementary Information

Supplementary Information 1.

Supplementary Information 2.

## Abbreviations

CFC: Chlorofluorocarbon
CH_4_: Methane
CML: Institute of environmental sciences
CO_2_: Carbon dioxide
Eq: Equivalent
FA: Fatty acids
FU: Functional units
GC: Gas chromatography
GHG: Greenhouse gas
GWP: Global warming potential
IA: Impact assessment
IC: Impact category
ILCD: International reference life cycle data system
MUFA: Monounsaturated fatty acid
NIST: Institute of standards and technology
PBS: Protaetia brevitarsis seulensis
PDF: Potentially disappeared fraction
SD: Standard deviation
SFA: Saturated fatty acid
kWh: Kilowatt hour
IPCC: Intergovernmental panel on climate change
ISO: International standardization organization
kg: Kilogram
LCA: Life cycle assessment
LCI: Life cycle inventory
MJ: Mega joule
N_2_O: Nitrous oxide
SO_2_: Sulfur dioxide
t: Tonne
