# Publisher Correction: Life cycle assessment of edible insects (*Protaetia brevitarsis seulensis* larvae) as a future protein and fat source

**DOI:** 10.1038/s41598-021-97513-y

**Published:** 2021-08-30

**Authors:** Amin Nikkhah, Sam Van Haute, Vesna Jovanovic, Heejung Jung, Jo Dewulf, Tanja Cirkovic Velickovic, Sami Ghnimi

**Affiliations:** 1 Department of Food Technology, Safety and Health, Faculty of Bioscience Engineering, Ghent University, Coupure Links 653, 9000 Ghent, Belgium; 2 Department of Environmental Technology, Food Technology and Molecular Biotechnology, Ghent University Global Campus, Incheon, South Korea; 3 Faculty of Chemistry, Centre of Excellence for Molecular Food Sciences, University of Belgrade, Belgrade, Serbia; 4 Research Group Sustainable Systems Engineering (STEN), Department of Green Chemistry and Technology, Ghent University, Coupure Links 653, 9000 Ghent, Belgium; 5 Serbian Academy of Sciences and Arts, Belgrade, Serbia; 6 CNRS, LAGEPP UMR 5007, Université Claude Bernard Lyon 1, 43 Bd 11 Novembre 1918, 69622 Villeurbanne, France; 7 ISARA Lyon, 23 Rue Jean Baldassini, 69364 Lyon Cedex 07, France

Correction to: *Scientific Reports* 10.1038/s41598-021-93284-8, published online 07 July 2021

In the original version of this Article, Sam Van Haute and Sami Ghnimi were omitted as corresponding authors. Correspondence and requests for materials should also be addressed to sam.vanhaute@ghent.ac.kr and sghnimi@isara.fr.

The original Article has been corrected.

