# Supplementary material for: Life cycle assessment of edible insects (Protaetia brevitarsis seulensis larvae) as a future protein and fat source
Source: Sci Rep. 2021 Jul 7;11:14030. doi: 10.1038/s41598-021-93284-8 (PMC8263613; doi:10.1038/s41598-021-93284-8)
Supplement: Supplementary file 1 — Supplementary Information 1. [file 41598_2021_93284_MOESM1_ESM.docx]

**Appendix A**

**Table A1.** Databases used to estimate the emissions from off-site operations.

| Activity | Database | Category | Unit | Activity UUID /Source |
| --- | --- | --- | --- | --- |
| Water-Unspecified natural origins | Input from nature | KR | m ^3^ | - |
| Electricity | ecoinvent 3 | KR | kWh | aa351ebf-456e-45c4-b6fd-7156f8d9c245 |
| Transport | ecoinvent 3 | GLO | t.km | 413c356e-677d-4676-b816-0c0b20768d7a_03bf1369-1eec-49d0-bc4b-8b29efa826b9.spold |
| Bio-waste treatment (composting) as the avoided product | ecoinvent 3 | CH | kg | ac9f6a67-bb3d-446a-9d31-e4d2f5279b3a_f43c95e7-40e6-4ba9-9ab1-d1bc10d3fa79.spold |
